# Needs of the population in Germany for information about health-related topics - Results from the KomPaS study

**DOI:** 10.25646/7146

**Published:** 2021-06-16

**Authors:** Kerstin Horch

**Affiliations:** Robert Koch Institute, Berlin, Department of Epidemiology and Health Monitoring

**Keywords:** HEALTH INFORMATION NEEDS, HEALTH CARE, PATIENT-FOCUSED, KOMPAS STUDY

## Abstract

Very few investigations have been conducted in Germany into the areas in which the population, including patients, lacks information about health-related issues. However, data from these areas provide crucial supplements to the descriptions and scientific analyses of health information behaviour that are more often available. Data on gaps in the population’s knowledge about health-related issues provide indications of health policy challenges. The Alliance for Health Competence, the German National Health Targets and the German National Health Portal, which was commissioned by the German Federal Ministry of Health, have all taken up this issue. The 2017 study ‘KomPaS: survey on communication and patient-safety’ was conducted by the Robert Koch Institute (RKI). The KomPaS study used the response categories ‘fairly well’ informed and ‘fairly poorly’ informed to assess how well-informed people feel when it comes to health-related issues. A comparison of the results from the supplementary survey conducted as part of the German Health Update (GEDA) 2009 and those of the KomPas study demonstrate varying degrees of improvement in the population’s level of health information in all areas over a period of almost ten years.

## Introduction

The population is very interested in health-related issues. This is also clear from the Fact sheet Searching for health information on the Internet – Results from the KomPaS study, which is published in this issue of the Journal of Health Monitoring. The Internet is an essential source of information and people are increasingly turning to it. However, traditional media such as television, radio and newspapers as well as conversations with doctors, family and friends are still important sources of information about health and illness. The relevance of the topic ‘searching for health information’ is, inter alia, reflected by other factors, increased levels of research and the rise in activities undertaken in this area. In addition to the large number of scientific studies published in recent years [1–5], health policy initiatives are also taking up this issue and its associated challenges [6–8]. These initiatives include the Alliance for Health Competence, the German National Heath Targets and the German National Health Portal, with the latter set up on behalf of the German Federal Ministry of Health. The National Action Plan for Health Literacy also discusses key aspects of this area and draws up a number of relevant measures.

Although a large number of descriptions and analyses focus on health information behaviour in different contexts, far fewer studies analyse gaps in the population’s (including the patient’s) knowledge and, therefore their information needs. However, studies that have investigated this issue, such as the German Health Update (GEDA) 2009 supplementary survey, which was conducted by the Robert Koch Institute (RKI), have identified a considerable need for information. This particularly applies to practical advice, and the information needed to make health care-related decisions. The study found that there are especially lacks of information in the population about the quality of health care services [2], about people’s satisfaction with various aspects of medical care (e.g. time, information, communication) [2], about exercising their rights and making complaints [2].

The RKI’s study ‘KomPaS: survey on communication and patient-safety’ took up this public health challenge with a number of specific questions. The aim was to determine the current information needs of the population in Germany. The study also compared its results with those of the GEDA 2009 supplementary survey in order to investigate trends.

**KomPaS study**

KomPaS: survey on communication and patient-safety

**Data holder:** Robert Koch Institute

**Objectives:** Describe informational needs, health literacy, patient safety, informed decision-making and physician’s counselling from the population’s point of view as part of patients’ information, decision-making and communication-related behaviour and the doctor-patient relationship.

**Survey method:** Computer-assisted telephone interview survey

**Study design:** Cross-sectional study

**Population:** German-speaking resident population in private households in Germany aged 18 or over

**Sampling:** Telephone sample comprising 60% landline and 40% mobile phone numbers

**Survey period:** May to September 2017

**Response rate:** 17.2%

**Sample size:** 5,053 participants

## Indicator

The KomPaS study and the GEDA 2009 supplementary survey asked participants to provide a self-assessment of how well informed they felt about various health-related issues (response categories: ‘fairly well’ informed and ‘fairly poorly’ informed). Data for the ‘health information needs’ indicator was collected using nine items: a set of questions about information on disease prevention and the different types of treatment available in the event of illness, two items about information issues relevant to patient-oriented health care (patient rights and who to contact about suspected medical errors) and items about their level of information concerning quality aspects in four health care areas.

The results (prevalences) of the KomPaS study are reported by sex, age group and socioeconomic status (hereinafter also referred to as social status) with 95% confidence intervals (95% CI). An indicator is used for social status that was developed using information provided by the respondents about their level of education, occupation and income. Statistical methods were used to test whether differences identified between groups were statistically significant. A statistically significant difference between groups is assumed if the corresponding p-value is less than 0.05. Statistically significant differences are explicitly stated. All analyses were carried out descriptively using the survey procedures provided by STATA SE 15.1 [9]. The analyses are based on data from a total of 5,053 participants aged 18 or older (56.7% women, 43.3% men). In order to ensure that the results can be viewed as representative, all calculations were carried out using a weighting factor that corrects deviations within the sample from the population structure (as of 31 December 2016).

## Results and discussion

**Figure 1:**
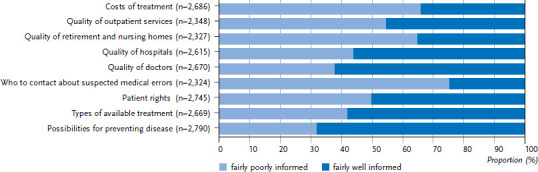
Percentage of women who feel ‘fairly poorly’ or ‘fairly well’ informed about selected health issues Source: KomPaS study (2017)

**Figure 2:**
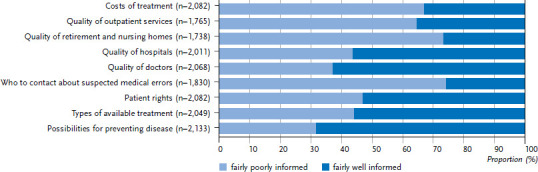
Percentage of men who feel ‘fairly poorly’ or ‘fairly well’ informed about selected health issues Source: KomPaS study (2017)

As Figure 1 and Figure 2 demonstrate, a large percentage of the population feels well informed about health information concerning disease prevention (68.4% overall). This applies equally to women and men (68.2% vs 68.6%). Evaluations undertaken by the KomPaS study demonstrate that older people feel better informed about health-related issues than younger people (this difference is statistically significant). This could be explained by the fact that people’s health tends to deteriorate with age and thus older people are presumably more interested in this topic. Although 58.2% of women and 56.3% of men surveyed report that they are ‘fairly well’ informed about the various forms of treatment available in case of illness, 42.8% of the overall population feels ‘fairly poorly’ informed about this issue. A relatively large percentage of the population articulates a need for information that would enable them to make their own decisions about health care (74.5% item ‘who to contact about suspected medical errors’, and 48.2% item ‘patient rights’). The desire for information about the quality of health care institutions should also be interpreted in this context: as sovereign users of health care services, people want to be able to make informed decisions for or against a particular health care provider. Women and men feel least well informed about the quality of retirement and nursing homes (68.8% in total) and about the quality of outpatient care services (59.2% in total). Women feel better informed (35.3% and 45.6%) about these quality aspects than men (26.9% and 35.6%). This difference is statistically significant. However, men feel somewhat better informed than women (52.5% and 56.2%) about the quality of doctors (63.1%) and the quality of hospitals (56.6%).

The analyses undertaken for the KomPaS study show a rise in the percentage of people who feel ‘fairly well’ informed about all items surveyed in 2009 (Table 1).

The subject areas in which the majority of the population felt ‘fairly poorly’ informed when the GEDA supplementary survey was carried out (2009) include who to contact about suspected medical errors, the quality of retirement and nursing homes and outpatient care services, as well as the cost of treatment. The results of the 2017 KomPaS study show that these issues are still the most relevant in terms of women’s and men’s greatest information needs in Germany.

Table 2 sets out results from the KomPas study with regard to the population’s information about the quality of health care services. Stratification by sex, age group, social status and health insurance (statutory/private) provides indications about population group-specific differences in information needs.

For many years, discussions have been ongoing about the lack of information on quality available to the population [10–13]. The introduction of the Hospital Report, the ‘White List’ and other measures [12] should improve the transparency of this type of information. In-depth analyses of whether this approach has been successful are currently lacking. However, various studies [11–15] demonstrate a significant need among users of the health care system and patients for information about quality. 81% of those surveyed in a study by the Bertelsmann Stiftung in 2018 [16] stated that more information about quality in the health care sector would help them to find a suitable service provider. At the same time, the respondents viewed the value of such data for quality development in the health care system as very high. One in four individuals is concerned that a lack of information might prevent them from finding the right doctor. Analyses of data from the KomPaS study supplement these results and indicate that quality-related information should take the needs of target groups into account (Table 2).

Figure 1 and Figure 2 demonstrate women’s and men’s levels of information about health-related issues. The significant differences between the sexes (see quality of retirement and nursing homes as well as outpatient care services) probably indicates the existence of sex-related differences due to the assumption of specific roles. Women are far more likely to take on the role of carer in the family than men [17, 18]. As such, they may also obtain information more frequently, and, consequently, be more likely to rate themselves as better informed. The differences between the age groups considered here are significant for all quality-related items and predominantly result in the expected picture of a lower level of information among younger age groups than among older ones. However, the very high proportion of older women and men who feel ‘fairly poorly’ informed about the quality of retirement and nursing homes is particularly striking. This difference is associated with social status: people in the higher social status group feel ‘fairly poorly’ informed about the quality areas considered compared with the medium and lower status groups. This result presumably reflects different expectations about the quality of care providers and the corresponding provision of information. This might also explain the differences between the information needs of people with statutory and private health care as it is likely that various socioeconomic differences exist between these two groups.

In summary, the results of the KomPaS study presented here show that there is still a great need for information among women and men about transparency in the health care system (e.g. information about quality and who to contact about suspected medical errors) and patients’ rights. However, the gaps in the other areas listed here also need to be filled by further improving health literacy and patient sovereignty. The results of the KomPaS study strengthen knowledge about the population’s information needs, and can contribute to shaping and developing the German National Health Portal.

## Key statements

- People have an urgent need for practical advice and information that can be used to make health care-related decisions.
- People particularly lack information about patient rights, who to contact in cases of suspected medical errors and about quality aspects and costs. However, this situation has improved since 2009.
- 68.4% of the population feels ‘fairly well’ informed when it comes to disease prevention; 57.2% feels ‘fairly well’ informed concerning information about the various forms of treatment available in cases of illness.
- Differences were identified by age, sex and socioeconomic status in terms of how well-informed people feel about health-related issues.

## Figures and Tables

**Table 1:** Percentage of people who feel ‘fairly well’ informed about various health-related issues (GEDA 2009 supplementary survey n=2,998 women and men; KomPaS study 2017 n=5,053 women and men) Source: GEDA supplementary survey (2009), KomPaS study (2017)

	GEDA 2009 supplementary survey	KomPaS 2017
Possibilities for preventing disease	63.0%	69.0%
Types of available treatment	54.5%	57.0%
Who to contact about suspected medical errors	12.0%	25.5%
Quality (doctors, hospitals, nursing homes, outpatient care services)	20%–35%	30%–60%
Treatment costs	20.0%	34.0%

**Table 2:** Percentage of the population that feels ‘fairly poorly’ informed about the quality of health care services by sex, age, socioeconomic status and health insurance Source: KomPaS (2017)

	Quality of doctors (N=4,738)		Quality of hospitals (N=4,626)		Quality of retirement and nursing homes (N=4,065)		Quality of outpatient nursing services (N=4,113)	
	% (n)	(95% CI)	% (n)	(95% CI)	% (n)	(95% CI)	% (n)	(95% CI)
**Sex**					***		***	
Women	37.5 (2,670)	(34.9–40.1)	43.8 (2,615)	(41.2–46.6)	64.7 (2,327)	(61.9–67.5)	54.4 (2,348)	(51.5–57.2)
Men	36.9 (2,068)	(34.2–3.7)	43.4 (2,011)	(40.5–46.3)	73.1 (1,738)	(70.2–75.8)	64.4 (1,765)	(61.2–67.4)
Total	37.2 (4,738)	(35.3–39.1)	43.6 (4,626)	(41.6–45.6)	68.8 (4,065)	(66.8–70.7)	59.2 (4,113)	(57.1–61.3)
**Age group**	***		***		***		***	
18–29 years	36.2	(30.4–42.4)	41.9	(35.7–48.2)	71.4	(64.2–77.6)	59.2	(52.3–65.8)
30–44 years	46.5	(41.8–51.2)	51.1	(46.2–55.9)	74.9	(70.3–79.1)	68.2	(63.1–72.9)
45–64 years	40.7	(38.0–43.4)	48.7	(45.9–51.6)	70.9	(68.0–73.6)	62.2	(59.2–65.1)
≥65 years	24.5	(22.0–27.1)	30.4	(27.7–33.2)	58.3	(55.0–61.6)	46.6	(43.4–49.9)
**Socioeconomic status**	***		***		***		***	
Low	30.0	(24.4–36.2)	36.9	(30.8–43.5)	64.1	(57.3–70.4)	48.3	(41.5–55.1)
Medium	35.1	(32.7–37.6)	42.3	(39.7–44.9)	66.9	(64.2–69.5)	57.7	(54.9–60.4)
High	46.3	(43.2–49.5)	50.5	(47.4–53.6)	76.0	(73.2–78.6)	70.9	(67.8–73.7)
**Health insurance**					*		***	
Statutory	36.6	(34.5–38.6)	43.0	(40.9–45.2)	68.2	(66.0–70.4)	57.1	(54.7–59.4)
Private	40.5	(35.7–45.4)	46.9	(42.1–51.7)	73.5	(68.7–77.8)	71.1	(66.1–75.6)

CI = confidence interval, ^*^p < 0.05, ^***^p < 0.001
